# Mutation Profiles of Ovarian Seromucinous Borderline Tumors in Japanese Patients

**DOI:** 10.3390/curroncol29050294

**Published:** 2022-05-18

**Authors:** Hiroki Sasamori, Kentaro Nakayama, Sultana Razia, Hitomi Yamashita, Tomoka Ishibashi, Masako Ishikawa, Seiya Sato, Satoru Nakayama, Yoshiro Otsuki, Ritsuto Fujiwaki, Noriyoshi Ishikawa, Satoru Kyo

**Affiliations:** 1 Department of Obstetrics and Gynecology, Shimane University School of Medicine, Izumo 6938501, Japan; sasamori@med.shimane-u.ac.jp (H.S.); raeedahmed@yahoo.com (S.R.); memedasudasu1103@gmail.com (H.Y.); tomoka@med.shimane-u.ac.jp (T.I.); m-ishi@med.shimane-u.ac.jp (M.I.); sseiya@med.shimane-u.ac.jp (S.S.); satoruky@med.shimane-u.ac.jp (S.K.); 2 Department of Obstetrics and Gynecology, Seirei Hamamatsu General Hospital, Hamamatsu 4308558, Japan; satoru@sis.seirei.or.jp; 3 Department of Organ Pathology, Seirei Hamamatsu General Hospital, Hamamatsu 4308558, Japan; otsuki@sis.seirei.or.jp; 4 Department of Obstetrics and Gynecology, Matsue Red Cross Hospital, Matsue 6908506, Japan; ritsu295@yahoo.co.jp; 5 Department of Pathology, Shonan Fujisawa Tokushukai Hospital, Fujisawa 2510041, Japan; noriyoshi.ishikawa@tokushukai.jp

**Keywords:** ovarian seromucinous borderline tumor, ovarian tumor, oncogene, tumor suppressor gene, mutation

## Abstract

Ovarian seromucinous tumors (SMBTs) are relatively rare, and their carcinogenesis is largely unknown. In this study, the molecular features of SMBTs in Japan are assessed. DNA was extracted from microdissected paraffin-embedded sections from 23 SMBT cases. Genetic mutations (*KRAS*, *BRAF*, *PIK3CA*, and *ERBB2*) were evaluated using Sanger sequencing. Immunohistochemistry for p53, ARID1A, and PTEN was also performed as a surrogate for the loss of functional mutations in these tumor suppressor genes. The prevalence of *KRAS, BRAF, PIK3CA,* and *ERBB2* mutations was 4.3% (1/23), 8.6% (2/23), 8.6% (2/23), and 17.3% (4/23), respectively. Overexpression or loss of p53 expression occurred in 26% (6/23), loss of ARID1A expression in 4.3% (1/23), and none of the cases showed expression of PTEN loss. These findings suggest that *KRAS/BRAF/PIK3CA* and *PTEN* mutations are rare carcinogenic events in SMBTs. The high frequency of positive p53 staining and a low frequency of loss of ARID1A staining suggests that SMBT carcinogenesis may be related to the alteration of *p53* rather than that of *ARID1A*. *ERBB2* oncogenic mutations may play an important role in the tumorigenesis of Japanese SMBTs.

## 1. Introduction

Previously, seromucinous borderline tumors (SMBTs) in the ovary were considered a subtype of mucinous borderline tumors (MBT) [1]. Although SMBTs are thought to be a subtype of MBTs, they are clinically similar to serous borderline tumors (SBTs) because both display papillary projection inside cystic spaces grossly and present hierarchical branching with broad fibrous stroma microscopically [2]. In 2014, the WHO presented several modifications to tumors that belong to female reproductive organs and classified SMBTs as a new category distinct from MBTs [3]. SMBTs are a novel morphological group that is thought to be derived from or associated with endometriosis (30–50%) in many cases, which is difficult to find in patients with SBTs or MBTs [4]. The frequent bilaterality of SMBTs and their association with endometriosis has been confirmed in several studies [5,6,7,8].

Few reports have suggested that numerous genetic alterations are associated with tumorigenesis in SMBTs [9,10,11]. The first comprehensive attempt was recently undertaken to investigate the molecular underpinning of SMBTs by applying next-generation sequencing. This report found that SMBTs’ signatures consisted of frequent somatic mutations in the *KRAS* (100%), *PIK3CA* (60.7%), and *ARID1A* (14.3%) genes, with *TERT* promoter mutations and DNA mismatch repair deficiencies being consistently absent [9]. Another paper confirmed that the loss of ARID1A expression, a surrogate for *ARID1A* mutations, has been reported in one-third of these tumors, a frequency similar to that seen in ovarian endometrioid carcinomas, supporting their close relationship [10]. A further study focused on *KRAS* and *PTEN* mutations of 16 samples reported that *KRAS* mutation was 69%, and no *PTEN* mutation was observed [11].

Thus, molecular biological analyses of SMBTs have revealed several molecular characteristics. However, these cases originated in Taiwan, Korea, and the United States [9,10,11]. The molecular profiling of SMBTs in Japanese patients is yet to be performed. This study aims to assess the molecular features of SMBTs in a Japanese population. The genetic alterations in *KRAS*, *BRAF*, *PIK3CA*, *ERBB2*, *PTEN*, *ARID1A*, and *p53* were retrospectively investigated to clarify the role of each gene in SMBT tumorigenesis.

## 2. Materials and Methods

### 2.1. Tumor Samples

Formalin-fixed, paraffin-embedded (FFPE) tissue samples from 23 SMBTs were used in this study. The samples were retrieved from the Department of Obstetrics and Gynecology, Shimane University Hospital, Seirei Hamamatsu General Hospital, and Matsue Red Cross Hospital between 2006 and 2019 in Japan.

The diagnoses were based on the conventional histopathological examination of sections stained with hematoxylin and eosin. Several pathologists categorized tumors according to the World Health Organization subtype criteria in each hospital. Tumors were staged according to the International Federation of Gynecology and Obstetrics classification system. All patients were primarily treated with unilateral/bilateral salpingo-oophorectomy and ± hysterectomy ± omentectomy ± pelvic lymphadenectomy. The resected specimen of each case was centrally reviewed by a gynecological pathologist (N.I.) and gynecologic oncologist (K.N.). The protocol for acquiring tissue specimens and clinical information was approved by the Institutional Review Board of Shimane University Hospital (IRB No. 20070305-1 and No. 20070305-2, version 10; last update, 8 December 2019). All the participants provided written informed consent. The study was conducted in accordance with the tenets of the Declaration of Helsinki and Title 45 (United States Code of Federal Regulations), Part 46 (Protection of Human Subjects), effective 13 December 2001.

### 2.2. Sample Processing and DNA Extraction

A gynecologic pathologist (N. I.) carefully selected representative FFPE blocks and identified the areas of SMBTs suitable for microdissection. Paraffin-embedded tissues were serially sectioned to a thickness of 10 mm. Genomic DNA for mutation analysis was extracted from FFPE samples using a commercially available kit (Qiagen, Inc., Valencia, CA, USA) as previously described [12].

### 2.3. Mutation Analysis

Extracted DNA was amplified by polymerase chain reaction (PCR) using primers for exon 2 of *KRAS*, exon 15 of *BRAF*, exons 9 and 20 of *PIK3CA,* and exon 20 of *ERBB2* using genomic DNA obtained from microdissected formalin-fixed paraffin-embedded tissue. The study focused on analyzing exons reported to harbor most mutations in each gene [13]. The primers used for amplification were: *KRAS*-Exon 2, forward primer 5′-TTAACCTTATGTGTGACATGTTCTAA and reverse primer 5′-AGAATGGTCCTGCACCAGTAA, *BRAF*-Exon15; forward primer 5′-TGCTTGCTCTGATAGGAAAATG and reverse primer 5′-AGCATCTCAGGGCCAAAAAT, *PIK3CA*-Exon9; forward primer 5′-GGGAAAAATATGACAAAGAAAGC and reverse primer 5′, *PIK3CA*-Exon 20; forward primer 5′-CTCAATGATGCTTGGCTCTG and reverse primer 5′-TGGAATCCAGAGTGAGCTTC, *ERBB2*-Exon 20; and forward primer 5′-CCATACCCTCTCAGCGTAC and reverse primer 5′-CGGAGAGACCTGCAAAGAG.

The thermal cycle profile for all gene amplification included one cycle at 95 °C for 30 s followed by 40 cycles at 55 °C and extension at 72 °C for 15 s. All polymerase-chain-reaction-amplified products were sequenced at Beckman Coulter (Danvers, MA, USA) and analyzed using the Mutation Surveyor DNA Variant Analysis Software (Tokyo, Japan). The pathogenicity of each mutation was confirmed using the Catalogue of Somatic Mutations in Cancer (COSMIC).

### 2.4. Immunostaining of ARID1A, p53, and PTEN

The expression levels of ARID1A, p53, PTEN, and ERBB2 were evaluated by immunohistochemical analysis (IHC). FFPE sections (3 μm thick) were dewaxed in xylene and hydrated in graded alcohol solutions. After antigen retrieval in a sodium citrate buffer, the slides were incubated overnight at 4 °C with antibodies at the following dilutions: 1:50 p53 (M7001; Dako, Carpinteria, CA, USA), 1:100 ARID1A (Sc-32761; Santa Cruz Biotechnology, Santa Cruz, CA, USA), 1:200 PTEN (138G6; Cell Signaling Technology, Danvers, MA, USA), and 1:200 ERBB2 (ab16901, abcam, Cambridge, UK). Two gynecologic oncologists (H. S. and K. N.), blinded to the clinicopathologic factors, independently evaluated the samples under a light microscope. The loss of ARID1A and PTEN expression in tumor cell nuclei was used as a surrogate for the presence of PTEN/ARID1A loss-of-function mutations [13]. Similarly, p53 immunoreactivity was used as a surrogate for the presence of p53 loss/overexpression of functional mutations. The assessment of ARID1A, p53, PTEN, and ERBB2 immunostaining was performed as described in our previous reports [14,15]. Briefly, ARID1A and PTEN immunoreactivity was scored by two investigators (H. S. and K. N.): 0, undetectable; 1+, weakly positive; 2+, moderately positive; and 3+, intensely positive. The loss of ARID1A or PTEN staining intensity (0+) was considered negative. Weak to moderate immunoreactivity was considered p53 normal expression. Strong and diffuse nuclear p53 immunoexpression or the complete absence of p53 staining was likely to indicate a p53 mutation.

A score of 2+ or 3+ was defined as ERBB2 overexpression, and the others were defined as low expression [16].

## 3. Results

The direct sequence analysis of 23 tumor specimens was performed to assess the mutation profiles of SMBTs. The patients’ clinical characteristics and mutation profile are summarized in Table 1 and Table 2. The age of the patients ranged from 18 to 83 years, with a mean and median age of 51.8 and 49 years, respectively. The majority of the patients (95.6%) were found in stage IA at diagnosis, and only one (4.34%) patient presented with stage IIA disease.

**Figure 1:**
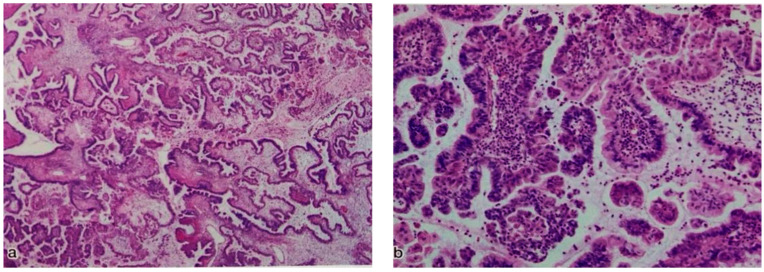
Representative histological characteristics of seromucinous borderline tumor (SMBT). (**a**) Low magnification (×10); (**b**) high magnification (×20).

The tumors’ size ranged from 3.7 to 31 cm, with a median size of 10 cm. The tumors involving the right ovary alone accounted for 56.6% (13/23), while those involving the left ovary alone accounted for 43.4% (10/23). No cases of bilateral ovarian SMBT were observed in this study. The patients initially underwent left/right or bilateral salpingo-oophorectomy ± hysterectomy ± omentectomy ± pelvic lymphadenectomy. Fertility preservation patients underwent unilateral salpingo-oophorectomy, and patients diagnosed with carcinoma by rapid intraoperative pathological diagnosis underwent total abdominal hysterectomy + bilateral salpingo-oophorectomy + omentectomy + pelvic lymphadenectomy. However, patients with carcinomas were diagnosed with SMBTs in the final postoperative diagnosis. All patients in the current study are still alive without disease, and their survival rate is 100%. Pathological evidence of endometriosis was confirmed in 13.1% (3/23) SMBTs. Figure 1 shows representative examples of the histological appearance of the SMBTs.

**Figure 2:**
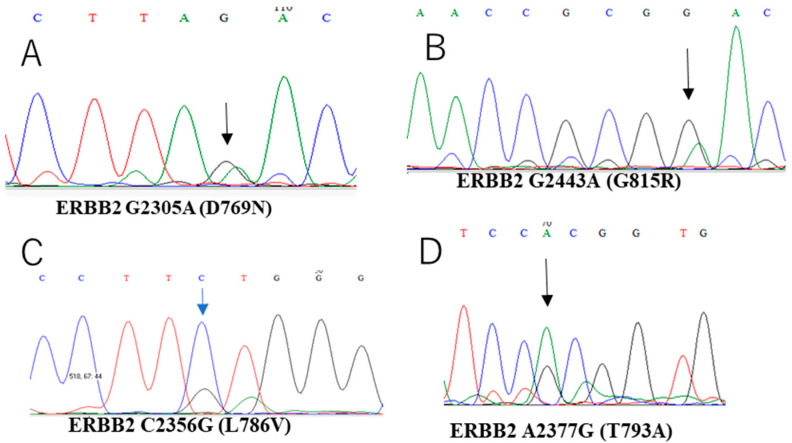
Chromatograms of ERBB2 mutation statuses in SMBTs. Each SMBT showed mutations (**A**) D769N (2305G>A), (**B**) G815R (2443G>A), (**C**) L786V (2356C>G), and (**D**) T793A (2377A>G) in the ERBB2 gene, respectively.

All 23 cases were assessed for mutations in *KRAS, BRAF, PIK3CA*, and *ERBB2*. The prevalence of *KRAS* (G12V), *BRAF* (V600E), *ERBB2* (D769N, T793A, G815R, and L786V), and *PIK3CA* (T544P) mutations was 4.3% (1/23), 8.6% (2/23), 17.3% (4/23), and 8.6% (2/23), repsectively (Table 3, Figure 2).

**Figure 3:**
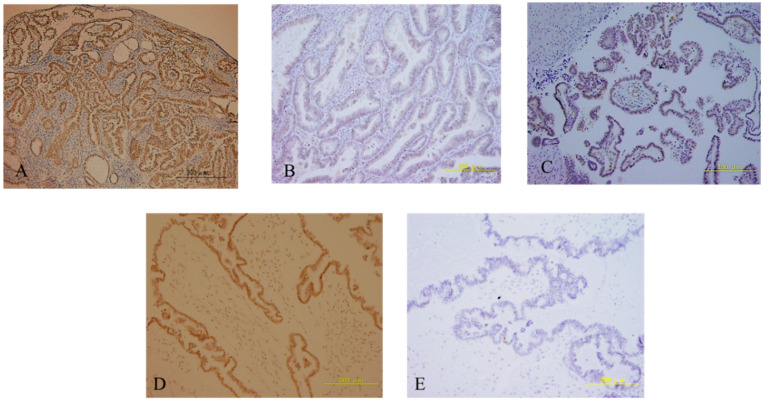

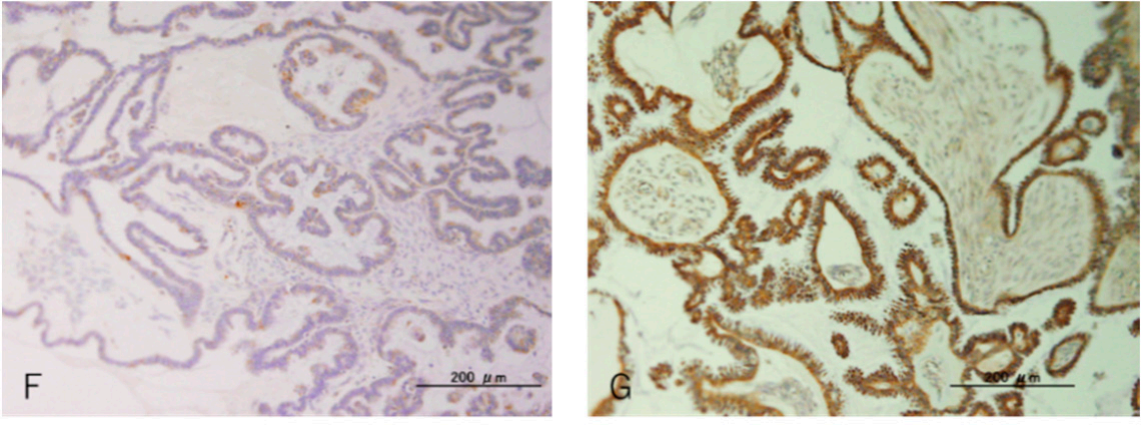
Representative immunohistochemical staining of ARID1A, PTEN, p53 and ERBB2 in SMBTs. Normal (**A**) and loss of ARID1A expression (**B**). Normal PTEN (**C**); overexpression (**D**) and loss of p53 expression (**E**); low (**F**) and overexpression (**G**) of ERBB2 in SMBTs.

The immunohistochemical expression of ARID1A, p53, and PTEN in all samples, as surrogates for these tumor suppressor gene mutations, was analyzed. The loss of ARID1A nuclear expression was observed in one case (4.3%). It was noted that p53 expression was undetectable in two cases, and overexpression was found in four cases (26.0%) (Figure 3). *PTEN* was expressed in all SMBTs, and none of them lost its expression, suggesting that no *PTEN* mutations were found (Figure 3). The ERBB2 protein expression level was evaluated, and all of the mutant cases of ERBB2 were overexpressed (Figure 3).

## 4. Discussion

Ovarian cancer accounts for more deaths than all other gynecologic malignancies [17] and typically affects postmenopausal women; about 12% are diagnosed under the age of 45, with 5% under the age of 35 years [18,19,20,21,22]. Treatment for ovarian cancer usually involves a combination of surgery and chemotherapy that can impact the ovaries anatomically or functionally. Patients, particularly young patients who want to preserve their fertility, diagnosed with malignant ovarian tumors, including SMBTs, experience anxiety, anger, sadness, and depression, severely impairing their lives [23]. For this reason, fertility-sparing treatment for SMBTs with psychological care is essential.

This study first described the molecular alterations of SMBTs in the Japanese population regarding the prevalence of mutations in *KRAS* (4.3%), *BRAF* (8.6%), *PIK3CA* (8.6%), *ERBB2* (17.3%), *ARID1A* (4.3%), *p53* (26%) and *PTEN* (0%). Very recently, Wu et al. [9] performed mutational analysis on 28 SMBTs in Taiwan and reported that somatic mutations in the *KRAS*, *PIK3CA*, *ARID1A*, and *PTEN* genes were 100, 60.7, 14.0, and 3.6%, respectively. They demonstrated that *KRAS* was mutated in all SMBTs, whereas *PIK3CA* mutations occurred frequently. Compared with their report, the current frequency of these alterations in these genes was much lower. Ethnic differences seem to contribute to the incidence and prognosis of cancers. It was previously discovered that the carcinogenesis signaling pathway in low-grade serous ovarian carcinoma was different between Japanese and Western populations. The mutation of *KRAS/BRAF* was observed in Western countries [24,25,26], whereas *PIK3CA* mutation was the main driver for Japanese low-grade serous carcinoma progression [27]. Racial/ethnic differences in breast cancer incidence and outcome have also been observed. African American women are diagnosed at an advanced stage with large tumors and a higher grade than those in White women [28,29]. Differences in pharmacokinetics and toxicity of anticancer drugs between Asian and White patients have been reported [30]. Allelic variants of genes encoding drug-metabolizing enzymes are expressed with different incidences in different ethnic groups [30]. This study thus hypothesized that the difference in prevalence might be due to a difference in genetic background between the Japanese population and other ethnicities or the difference in methods between direct sequencing and next-generation sequencing. SMBTs are associated with endometriosis, and previous studies have identified this in 45–71% of patients [5,6,7,8,9], including three cases in which endometriosis was directly contiguous with SMBTs [31]. The current frequency of SMBTs with endometriosis is much lower than that reported in previous reports [5,6,7,8,9]. Therefore, it may also be because the frequency of SMBTs with concurrent endometriosis is significantly lower than that reported by Wu et al. [9].

In 2014, the World Health Organization defined SMBTs as a new histological subtype of ovarian carcinoma, but seromucinous carcinoma was not defined in endometrial carcinoma [3]. “Seromucinous Carcinoma” has been removed from the 2020 5th edition of the WHO Classification of Female Genital Tumors because this is a poorly reproducible diagnosis. Moreover, there is significant morphological overlap with endometrioid carcinoma [32]. Therefore, seromucinous carcinoma is considered a subtype of endometrioid carcinoma [33,34]. *ARID1A* is a tumor suppressor gene that is frequently mutated in endometriosis-related ovarian neoplasms, including clear cell and endometrioid carcinoma. Previously, a somatic mutation in *ARID1A* has been found in 46–57% of ovarian clear cell carcinomas [35,36], 30% of ovarian endometrioid carcinomas [34], and 40% of uterine endometrioid carcinomas [37]. Among 24 SMBTs, loss of ARID1A expression in 8/24 (33%) cases and one case with loss of ARID1A expression in synchronous endometriosis were reported [10]. A significant number of SMBTs that exhibit loss of expression of ARID1A and their common association with endometriosis reveal that these tumors are closely related to endometrioid carcinomas. However, in the current study, the loss of ARID1A was only 4.3%, which was lower than that in previous reports [5,6,7,8,9,10]. In addition, immunohistochemical staining for p53 has been found positive (mutant p53) in 26.0% (6/23) of SMBTs. Previously, mutational profiles of endometriosis-related ovarian neoplasms (ERONs) were constructed [38]. It was observed that *ARID1A* mutation was 95%, while *p53* was 36.8%, suggesting that the occurrence of some ERONs based on *p53* alterations was similar to that in the current findings in SMBTs.

Molecular alterations in SMBTs strikingly differ from those of other borderline ovarian tumors (serous and mucinous) in the ovary. Previously, Sanger sequencing was performed to determine the molecular mechanisms involved in the tumorigenesis of MBTs and SBTs. *BRAF* (40%) and *KRAS* (20%) mutations are the most frequent genetic alterations in MBTs, whereas *PIK3CA* (63.6%) mutations are the most responsible for the tumorigenesis in SBTs [27,39]. In contrast, the current study observed that *ERBB2* was the highest prevalent mutant oncogene (17.3%) in SMBTs. This finding suggests that each borderline tumor has distinct molecular mechanisms of carcinogenesis.

The strength of this study is that it is the first molecular analysis of Japanese SMBTs. However, this study had several limitations. First, this study included a small number of patients as SMBTs are rare tumors; therefore, concrete conclusions cannot be drawn. Hence, further investigation with a larger study population is essential. Second, genetic mutations were identified using Sanger sequencing; therefore, the types of gene mutations assessed were limited. Additionally, IHC for p53, ARID1A, and PTEN was used as a surrogate marker for these tumor suppressor genes underlying the molecular derangements in SMBTs. Sanger sequencing will be necessary to confirm the IHC results in the future. Furthermore, next-generation sequencing is also needed to determine the comprehensive molecular mechanisms underlying the progression of SMBTs in Japanese patients.

The current findings suggest that alterations in *KRAS/BRAF/PIK3CA* and *PTEN* are rare carcinogenic events in Japanese SMBTs. The high frequency of positive p53 staining and a low frequency of loss of ARID1A staining suggests that the carcinogenesis of SMBTs could be related to the alteration of *p53* rather than that of *ARID1A*. The fact that the *ERBB2* oncogenic mutation is the highest event suggests its important role in the tumorigenesis of Japanese SMBTs.

## Figures and Tables

**Table 1:** Clinical information of the 23 SMBTs.

Case NO.	Age	Stage	Site of Tumor	Tumor Size (CM)	Surgical Type	Endometriosis
1	57	IA	Right	17	BSO+TAH+omentechtomy+pelviclymphadenectomy	No
2	66	IA	Right	15	BSO+omentechtomy	No
3	18	IA	Left	10	LSO	No
4	49	IA	Left	10	BSO+TAH+omentechtomy	No
5	28	IA	Right	17	RSO+omentechtomy	No
6	77	IA	Right	6	BSO+TLH	No
7	37	IA	Left	6.5	BSO+TAH+omentechtomy	No
8	41	IA	Right	9	BSO+TAH+omentechtomy	Yes
9	37	IA	Right	19	BSO+TAH+omentechtomy+pelviclymphadenectomy	No
10	47	IA	Left	22	BSO+TAH+omentechtomy	No
11	76	IA	Right	ND	RSO	No
12	54	IA	Left	ND	LSO	No
13	45	IA	Right	ND	BSO	Yes
14	58	IA	Left	ND	BSO+TAH	Yes
15	31	IA	Right	21	RSO	No
16	67	IIA	Left	9.5	BSO+TAH+pelviclymphadenectomy	No
17	47	IA	Right	12	RSO	No
18	26	IA	Right	31	RSO	No
19	83	IA	Left	3.7	BSO+TAH	No
20	72	IA	Left	9.2	BSO	No
21	76	IA	Right	5	BSO+TAH+omentechtomy	No
22	40	IA	Right	30	RSO	No
23	60	IA	Left	8.8	LSO	No

BSO: Bilateral salpingo-oophorectomy; LSO: left salpingo-oophorectomy; RSO: right salpingo-oophorectomy; TAH: total abdominal hysterectomy, ND: no data available.

**Table 2:** Mutation profile of 23 SMBTs.

Case NO.	p53 (IHC)	ARID1A (IHC)	PTEN (IHC)	*KRAS*	*BRAF*	*PIK3CA-E9*	*PIK3CA-E20*	*ERBB2*	ERBB2 (IHC)
1	Normal	Normal	Normal	-	-	-	-	D769N. c.2305G>A	High
2	Loss	Normal	Normal	-	-	-	-	-	High
3	Loss	Normal	Normal	-	-	-	-	-	High
4	Overexpression	Normal	Normal	G12V, c.35G>T	-	-	-	-	Low
5	Overexpression	Normal	Normal	-	V600E, c.1799T>A	-	-	-	High
6	Normal	Normal	Normal	-	-	-	-	-	High
7	Normal	Normal	Normal	-	-	-	-	-	ND
8	Normal	Normal	Normal	-	-	-	-	-	Low
9	Normal	Normal	Normal	-	-	-	-	-	Low
10	Normal	Normal	Normal	-	-	-	-	-	Low
11	Normal	Normal	Normal	-	-	-	-	-	High
12	Normal	Loss	Normal	-	-	-	-	-	Low
13	Normal	Normal	Normal	-	-	-	-	-	ND
14	Normal	Normal	Normal	-	V600E, c.1799T>A	-	-	T793A, c.2377A>G	High
15	Normal	Normal	Normal	-	-	-	-	-	High
16	Overexpression	Normal	Normal	-	-	-	-	-	Low
17	Normal	Normal	Normal	-	-	-	-	-	Low
18	Normal	Normal	Normal	-	-	-	-	G815R, c.2443G>A	High
19	Normal	Normal	Normal	-	-	-	-	L786V, c.2356 C>G	High
20	Normal	Normal	Normal	-	-	-	-	-	High
21	Normal	Normal	Normal	-	-	T544P, c.1630A>C	-	-	High
22	Overexpression	Normal	Normal	-	-	T544P, c.1630A>C	-	-	High
23	Normal	Normal	Normal	-	-	-	-	-	High

ND: no data available.

**Table 3:** Mutation frequency in the 23 SMBTs.

Gene.	Frequency of Genetic Alteration
*KRAS*	4.3% (1/23)
*BRAF*	8.6% (2/23)
*PIK3CA*	8.6% (2/23)
*ERBB2*	17.3% (4/23)
*ARID1A*	4.3% (1/23)
*p53*	26% (6/23)
*PTEN*	0% (0/23)

## Data Availability

The data presented in this study are available upon request from the corresponding author (K.N.).
